# Correction: Sperm whales habituate to research vessels engaged in photoidentification

**DOI:** 10.1371/journal.pone.0353342

**Published:** 2026-07-07

**Authors:** Hal Whitehead, Christine M. K. Clarke, Ana Eguiguren

S1 Dataset was uploaded incorrectly. Please see the correct S1 Dataset below.

## Supporting information

S1 Data Data for analysis in Excel file. Sheets: All previous years; Just year in question; Data for social units; Explanation.
(XLSX)
