# Supplementary material for: Sperm whales habituate to research vessels engaged in photoidentification
Source: PLoS One. 2026 May 14;21(5):e0348681. doi: 10.1371/journal.pone.0348681 (PMC13175581; doi:10.1371/journal.pone.0348681)
Supplement: S1 Dataset — (PDF) [file pone.0348681.s001.pdf]

# Supporting Information to “Sperm whales habituate to research vessels engaged in photoidentification”

Hal Whitehead[1], Christine M. K. Clarke[1], Ana Eguiguren[1]

‘[1] Department of Biology, Dalhousie University, Halifax, Nova Scotia, Canada

## Data

The data for the analyses are in the attached Excel file, which has four sheets:

‘All previous years’: Data for most analyses, which considers photoidentifications from previous years when calculating independent variables.

‘Just year in question’: Data for analyses which consider only photoidentifications from the year being considered when calculating independent variables (results in Table A1).

‘Data for social units’: Data for identified social units (used to construct Figure 5).

‘Explanation’: Explanation of fields in Excel sheets.

## Additional dependent variable: proportion of very poor photographs

The proportions of very poor photographs ($Q$=1-2) on each day, $q_{12}$, showed similar relationships as $q_{13}$ with the dependent variables *api*, *ppi*, *cpi* (Fig A2; compare with Fig 3), although as the numerators in the $q_{12}$ proportions were necessarily smaller than for $q_{13}$, $q_{12}$ was a less precise measure. $q_{12}$ fell from $q_{12}$=0.103 (95%c.i. 0.089-0.119) for whales with no previous experience of the research vessel, to $q_{12}$=0.099 (95%c.i. 0.080-0.123) when all animals had experience (*ppi*=1), and $q_{12}$=0.077 (95%c.i. 0.057-0.104) when cumulative experience was at its highest (see Fig A2).

## Additional dependent variable: mean range to photographed animals

For mean ranges to photographed animals, $r_{mean}$, the best fitting model included none of the independent variables, instead representing a quadratic function of the control variable *sdd* (the number of days since the start of the study; see Fig A1). Thus, there is little evidence that this mean range at which photographs were taken varied with the experience of the whales with the

research vessel (Fig A3). As photographs were occasionally taken of quite distant or close flukes (e.g. >400m or <25m), the mean ranges will be generally less precise than the median ranges, and less likely to show changes in whale behaviour.

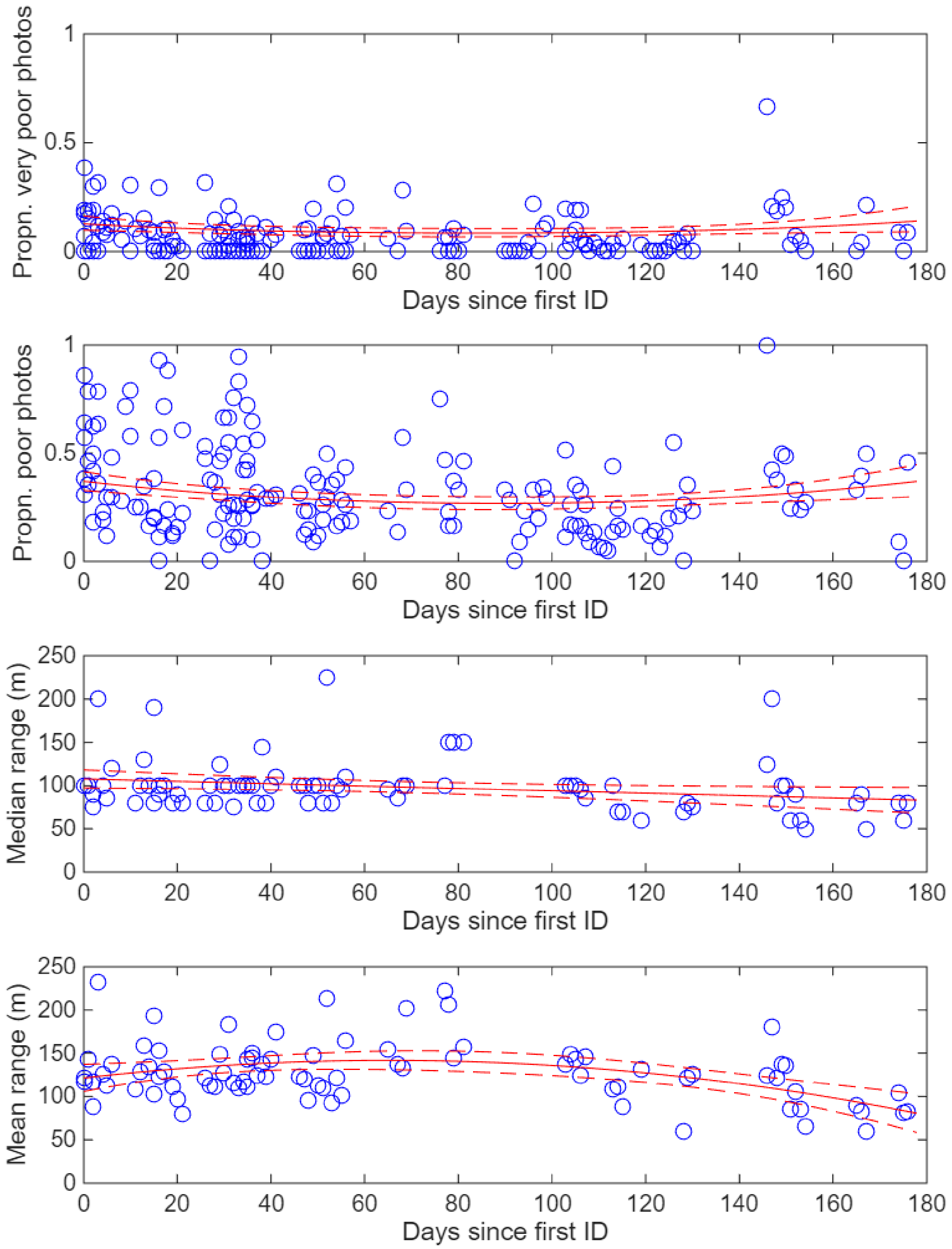


**Fig A1. The proportion of very poor photographs ($q_{12}$), poor photographs ($q_{13}$), as well as the median and mean ranges to photographed flukes ($r_{med}$, $r_{mean}$) with days since the start of that year's field study.** Also shown are prediction lines and 95% confidence intervals from generalized linear or quadratic models (whichever had lower AIC) with binomial error for $q_{12}$ and $q_{13}$, and normal error for $r_{med}$ and $r_{mean}$.

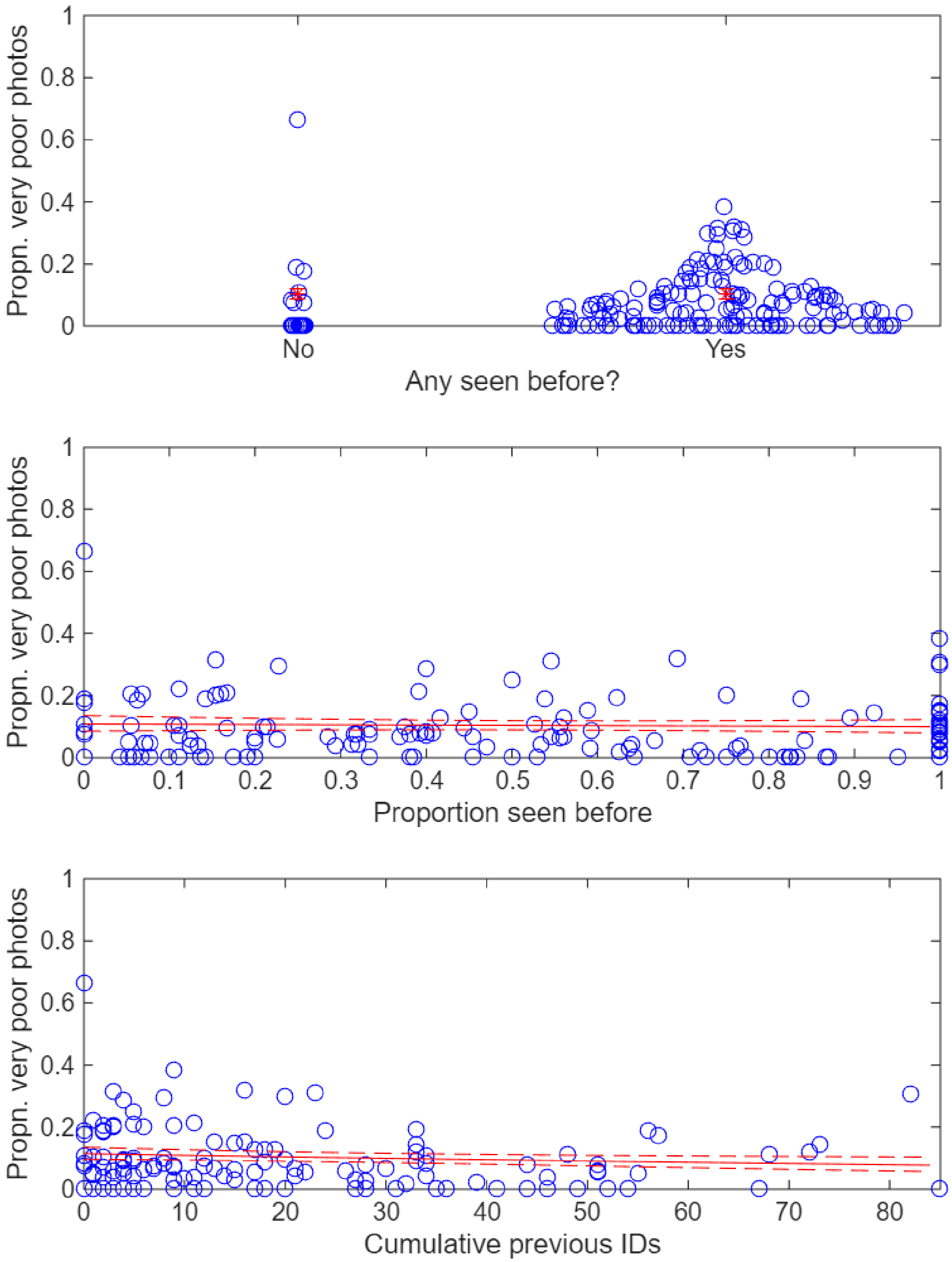


**Fig A2. Proportion of very poor photographs ($q_{12}$) on each day (raw values) against the three independent variables (*api, ppi* and *cpi*).** Also shown are a best fit line (plus 95% confidence intervals for the line) from a generalized linear mixed effect models with binomial error.

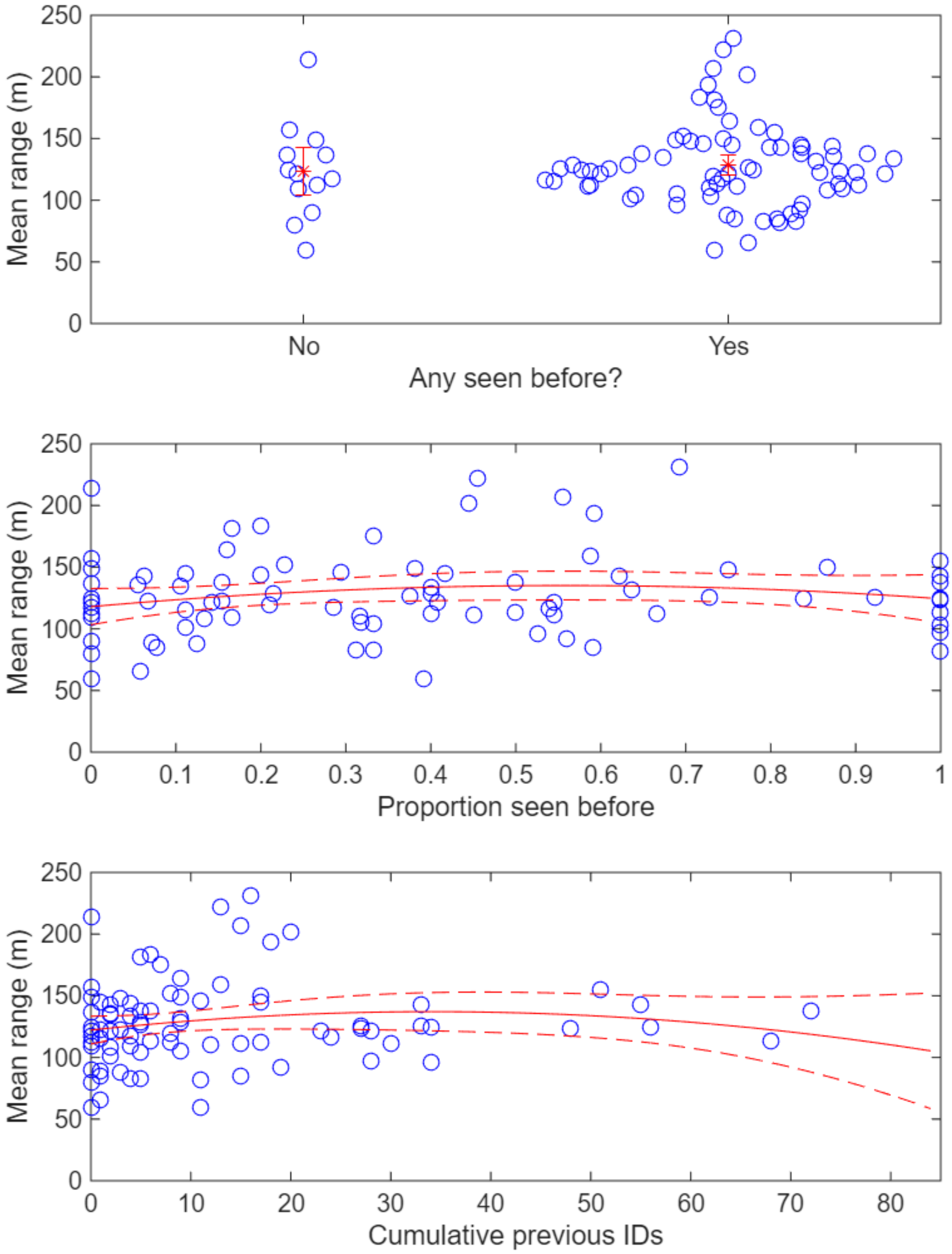


**Fig A3. Mean range to photographed animals ($r_{mean}$) on each day (raw values) against the three independent variables (*api*, *ppi* and *cpi*).** Also shown are a best fit line (plus 95% confidence intervals for the line) from a generalized linear model, although a model with control variables only, fit better.

**Table A1. With the proportion of poor photographs ($q_{13}$) as the dependent variable, this compares the fit of models to independent and control variables when the independent variables (*api*, *ppi*, *cpi*) were calculated using prior identifications from just the current year (left) or current and previous years (right, as in main text).**

| | Just current year | | | | Current and previous years | | | | AIC(CY)- |
|---|---|---|---|---|---|---|---|---|---|
| Model | AIC | ΔAIC | Coeff | P | AIC | ΔAIC | Coeff | P | AIC(CPY) |
| 1 + (1\|*Year*) | 550.09 | 61.13 | | | 550.09 | 64.62 | | | |
| *sdd* + (1\|*Year*) | 546.40 | 57.44 | | | 546.40 | 60.93 | | | |
| $sdd + sdd^2$ +(1\|*Year*) | 533.37 | 44.42 | | | 533.37 | 47.90 | | | |
| *api* + (1\|*Year*) | 525.78 | 36.83 | -0.401 | 0.000 | 543.47 | 57.99 | -0.222 | 0.046 | |
| *api* + *sdd* + (1\|*Year*) | 526.25 | 37.30 | -0.001 | 0.361 | 541.96 | 56.49 | -0.001 | 0.092 | |
| $api + sdd + sdd^2$ + (1\|*Year*) | 518.45 | 29.50 | -0.008 | 0.004 | 529.72 | 44.24 | -0.010 | 0.000 | -11.26 |
| *ppi* + (1\|*Year*) | 498.00 | 9.05 | -0.668 | 0.000 | 498.62 | 13.15 | -0.715 | 0.000 | |
| $ppi + ppi^2$ +(1\|*Year*) | 498.61 | 9.66 | | | 501.04 | 15.57 | | | |
| *ppi* + *sdd* + (1\|*Year*) | 493.78 | 4.82 | -0.002 | 0.019 | 492.85 | 7.38 | -0.002 | 0.011 | |
| $ppi + ppi^2 + sdd$ +(1\|*Year*) | 495.85 | 6.89 | | | 495.22 | 9.75 | | | |
| $ppi + sdd + sdd^2$ + (1\|*Year*) | 488.96 | 0.00 | -0.008 | 0.004 | 485.47 | 0.00 | -0.010 | 0.001 | 3.48 |
| $ppi + ppi^2 + sdd + sdd^2$ +(1\|*Year*) | 491.00 | 2.04 | | | 488.01 | 2.54 | | | |
| *cpi* + (1\|*Year*) | 491.49 | 2.54 | -0.011 | 0.000 | 491.70 | 6.22 | -0.009 | 0.000 | |
| $cpi + cpi^2$ +(1\|*Year*) | 493.58 | 4.63 | | | 494.31 | 8.83 | | | |
| *cpi* + *sdd* + (1\|*Year*) | 489.15 | 0.20 | -0.002 | 0.067 | 487.59 | 2.12 | -0.002 | 0.028 | |
| $cpi + cpi^2 + sdd$ +(1\|*Year*) | 491.25 | 2.30 | | | 489.83 | 4.36 | | | |
| $cpi + sdd + sdd^2$ + (1\|*Year*) | 489.09 | 0.13 | -0.005 | 0.111 | 486.86 | 1.39 | -0.006 | 0.051 | 2.22 |
| $cpi + cpi^2 + sdd + sdd^2$ +(1\|*Year*) | 491.21 | 2.26 | | | 489.06 | 3.58 | | | |

For each model and each scenario for calculating the independent variables ("Just current year", or "Current and previous years"), the table gives AIC, ΔAIC, the coefficient of the independent variable when it was fitted as a linear term, and its P-value. For each scenario, the best fitting model (minimum AIC) is highlighted in green, and other models with substantial support (ΔAIC <2.0) are highlighted in yellow. The table only shows models including *Year* as a random control variable, as these fit substantially better than models without *Year*. The final column gives the difference in AIC for the best models for each independent variable between the "Just

current year" and "Current and previous years" scenarios (AIC(CY)-AIC(CPY)). If memory of experience of the research vessel in previous years is important in determining behaviour, this difference should be positive.
