# Supplementary material for: Sperm whales habituate to research vessels engaged in photoidentification
Source: PLoS One. 2026 May 14;21(5):e0348681. doi: 10.1371/journal.pone.0348681 (PMC13175581; doi:10.1371/journal.pone.0348681)
Supplement: Attachment — Submitted filename: Response_to_Reviewers_auresp_2.docx [file pone.0348681.s004.docx]

Note added 9 April:

In the latest review process we received no new feedback and that all previous editorial concerns have been addressed.

See email exchange with Kit Stokes, Associate Peer Review Operations Specialist

We are grateful to the reviewers for their careful and constructive reviews of our paper. Their comments have led to improvements. Below we (in red font) respond to their comments (in yellow highlight) with line numbers referring to the revised text:

Reviewer #1:

However, the manuscript could be improved by offering a deeper interpretation of the control variable sdd, which showed a U-shaped quadratic effect where poor photographs decreased for the first 80 days but rose toward the end of the season. While the authors statistically control for this, the Discussion lacks a biological or operational hypothesis for this late-season reversal—potentially addressing whether this indicates observer fatigue or a shift from habituation to sensitization after prolonged exposure.

We expanded the consideration of *sdd* in the Results, and added a paragraph in the Discussion:

(Results; LL 167-175) “The control variable *sdd*, the number of days since the start of field season, was included as a quadratic term in the best fitting models for both dependent variables. The importance of *sdd* was relatively small compared with each of the independent variables for *q_13_*, the proportion of poor-quality photoidentifications (Table 2). However, on its own, *sdd* explained *r_med_*, the median range, almost as well as the independent variables that measure whale experience of the research vessel (Table 2). Given the experience of the whales to the research vessel, the effectiveness of the photoidentification process (in terms of the proportion of good quality photoidentifications) seems to generally increase during the first 2-3 months of a field season, and decline thereafter (S1 Fig).”

(Discussion; LL 221-228) “The quadratic relationship between *sdd*, the number of days since the start of field season, and the efficiency of the photoidentification process could result from systematic changes in the photoidentification methods or crew abilities or whale behavior as field seasons progressed. Changes in whale behavior are more likely related to whale experience of the research vessel (covered by the dependent variables) than the length of the field season, and “new whales” were photographed at all stages of each field season. Thus, we suspect that the changes in phototidentification efficiency over the field season are more likely due to crew members learning in the early days, and becoming fatigued after several months.”

However, the discussion regarding ‘habituation transfer’ to other vessel types warrants expansion. Given that the data set concludes in 1999 and relies on auxiliary sailing vessels, the authors should discuss how these findings translate into modern research environments involving high-speed motorized vessels or drones, which may elicit different behavioral responses. Explicitly contrasting the ‘benign’ nature of their specific vessel protocols (slow approach, no biopsy) against more invasive modern techniques would strengthen the external validity of their conclusion that photo-ID is largely harmless.

We think that an “explicit contrast” of the effects of different research vessels and field protocols on the subjects is beyond the scope of this paper. We have, however, added in Discussion (LL 298-303): “Field research on sperm whales, and other cetaceans, is becoming more diverse, with field scientists using vessels that are often much faster than our auxiliary sailing vessel, often louder (for instance when using high-powered outboard motors), but sometimes quieter (electric motors [e.g., 31]). Techniques are changing, with photoidentifications and other data being obtained from drones and in other ways [e.g., 32]. Such field techniques will likely have quite different impacts on the animals than those assessed here.”

Figure 1 (the vessel following a whale) and Figure 2 (examples of Q-values) would be more effective if combined into a single ‘Methods Plate’. Currently, Figure 2 displays photographic quality ranging from Q=2 to Q=5. However, the study’s primary dependent variable is q13 , the proportion of ‘poor’ photographs defined as Q = 1–3. Figure 2 should include an example of a Q=1 (‘extremely poor’) photograph to aid the reader in visually understanding what constitutes an ‘evasive’ or ‘failed’ data point.

We do not see value in combining Figures 1 and 2. They do refer to the same Methods section of the paper, but to different aspects: obtaining photoidentifications, and measuring photographic quality, respectively. We do agree that a Q=1 image is needed in Figure 2, and have added this.

Furthermore, the caption for the vessel image (Figure 1) should explicitly state the distance and orientation shown (e.g., 50m behind, matching speed) to visually confirm the ‘benign’ protocol described in the text.

These photographs are from 40 years ago. We do not have these data.

Figures 3 and 6
For Figures 3 and 6, which display the effects of experience (ppi, api, cpi) on photo quality (q13) and range (rmed), the presentation of the variable needs adjustment. The text defines [api] as a binary measure (whether any animals had been identified previously: yes/no). Plotting a continuous regression line with confidence intervals for a binary variable can be visually misleading. It would be more rigorously presented as a box-and-whisker plot or a violin plot comparing the two states (Experience vs. No Experience).

In this case, we like the “swarmplot” better than a box or violin plot, as it displays all the data rather than just a summary. We have followed the reviewer’s suggestion and removed the regression line for the binary *api*.

Additionally, for the continuous variables (ppi and cpi), the authors should clarify in the captions whether the data points plotted are raw values or partial residuals controlled for the ‘Year’ and ‘day of season’ (sdd) variables, as the text notes that has a non-linear U-shaped effect that was statistically controlled for.

These are raw values. And we have added this to the captions, so, for instance: “Figure 3. Proportion of poor photographs (*q*_13_) on each day (raw values) against the three independent variables...”

Figure 4
The figure caption should acknowledge that the wide confidence intervals in 1995 and 1999 indicate low predictive power due to small sample sizes.
The regression line for the 1995 and 1999 panels are visually weak due to massive confidence intervals. The text claims results are ‘quite consistent within all primary study years’, but Figure 4 visually contradicts this for 1995/1999 by showing lack of precision. The authors should soften the claim of consistency or explicitly note the increased uncertainty shown in these specific panels.

We have added to the caption of Figure 4: “Data are quite few for 1995 and 1999, and poorly balanced for 1999.”

We have also explicitly noted the uncertainty for 1999 panels in the Results (L 202): “; although data are unbalanced for 1999”.

Supplementary Figure S1
The text describes a "U-shaped quadratic effect" where photo quality initially improves but then deteriorates after 80 days into a season. Given that this finding complicates the ‘habituation’ narrative (potentially suggesting observer fatigue or late-season sensitization), the authors should consider moving Figure S1 from the Supplementary Materials into the main manuscript. Visualizing this reversal is important for the transparency of the study, as it justifies the inclusion of the term in the final statistical models.

Although the term *sdd* is included in the final statistical model (as noted in Table 2), its effect is quite small. Therefore, to focus the main text on the primary results, we prefer leaving Figure S1 in the Supporting Information.

Reviewer #2:
1 - Lines 199-208, first paragraph of Discussion: of explanatory mechanisms for habituation-type response, the authors are quick to dismiss mechanisms other than a learning-based habituation response on grounds that the individuals in the study have been re-sighted over many years, and a quiet boat. This feels legit to a reader familiar with Whitehead et al’s long history of work with this population but I feel deserves stronger support, ie how do we known that resight was not biased to individuals amenable to habituation?

The individuals (social units) that were repeatedly studied showed a clear habituation response. However, the reviewer makes the interesting suggestion that there may have been other individuals (social units) that showed a sufficiently aversive response to the research vessel that they were not resighted. This is possible but unlikely, because the research vessel typically detected (usually acoustically) and approached groups of sperm whales from ranges of several km, likely before the whales will have been aware of the vessel. The groups were then tracked, usually quite easily. Thus, to avoid interactions with the research vessel, a social unit would normally have had to actively avoid our large study area around the Galápagos Islands (of the order of 60,000km^2^), an area with generally low anthropogenic impact. This seems unlikely.

We have added to the Discussion (LL 242-248): “Our results do not rule out the possibility that some individuals, or more probably social units, might not have been repeatedly followed due to their aversion to the research vessel. However, the research vessel typically detected (usually acoustically) and approached groups of sperm whales from ranges of several km, likely before the whales will have been aware of the vessel, and groups were followed fairly easily [18]. Thus, to minimize being tracked by the research vessel, social units would have to avoid the study area, which was large (tens of thousands of sq km) and had relatively little anthropogenic impact. Such displacement seems unlikely.”

2 - Understood the data have a great deal of variation limiting power to interpret multi-year habituation but is there an opportunity for discussion of inter- vs intra-year habituation? Is there any indication of de-habituation between seasons? Can anything be learned about the habituation process, ie individuals become more tolerant of one well behaved vessel during each season but are behaviors changing over lifetimes of these animals? I’d think Gero’s work in Dominica has seen whether or not this is so, though am not aware of if there’s any publications on the topic.

We did look at this, by comparing the fit of models including and not including experience from previous years (LL 212-218). Unfortunately, the results were inconclusive, so we have no indication of de-habituation between seasons. The reviewer is correct in that Gero’s research in Dominica, with many resights of known individuals within and between field seasons, should have power to detect such effects, but, to our knowledge, this analysis has not been undertaken.

3 - Could the research be biased by habituation? Better quality photos of (presumably) more relaxed, less behavior state-shifting family groups is likely a positive but are there any blindspots that we are missing?

We think it unlikely that the research is biased by habituation. Even though it was more difficult to obtain high quality photographs on days following naïve animals, we still individually identified many of the whales present.

Very minor edits:
Line 126-135 - seems logical to reorder to api -> ppi -> cpi to scalę from simple to complex measure of familiarity (here and in subsequent mentions of the three metrics)

Good point. We have done this in the text, as well as the figures and tables.

Line 235 - add comma: the more experience, the less aversion

Done
